# Chronopotentiometric Evaluation of Ionization Degree and Dissociation Constant of Imidazolium-Based Ionic Liquid [C_6_Meim][NTf_2_] in Polymeric Plasticized Membranes

**DOI:** 10.3390/membranes12020130

**Published:** 2022-01-21

**Authors:** Nadezhda V. Pokhvishcheva, Elizaveta K. Gigiadze, Andrey V. Kalinichev, Alexandr V. Ievlev, Konstantin V. Tyutyukin, Maria A. Peshkova

**Affiliations:** 1 Institute of Chemistry, Saint Petersburg State University, 7/9 Universitetskaya nab., 199034 St. Petersburg, Russia; st040584@student.spbu.ru (N.V.P.); st055887@student.spbu.ru (E.K.G.); a.v.kalinichev@spbu.ru (A.V.K.); 2 Faculty of Physics, Saint Petersburg State University, 7/9 Universitetskaya nab., 199034 St. Petersburg, Russia; a.ievlev@spbu.ru (A.V.I.); k.tyutyukin@spbu.ru (K.V.T.)

**Keywords:** polymeric membranes, ionic liquids, diffusion coefficients, plasticizer, ion-selective sensors, ionization degree, ion pairs, polarity, chronopotentiometry

## Abstract

Ionic liquids (ILs) have a wide variety of applications in modern electrochemistry due to their unique electrolytic properties. In particular, they are promising candidates as dopants for polymeric membranes in potentiometric sensors and liquid-junction free reference electrodes. However, the effective use of ILs requires a comprehensive understanding of their electrolytic behavior in the polymeric phase. We report here the exploration of the electrolytic and diffusion properties of IL 1-hexyl-3-methyl-1H-imidazol-3-ium bis[(trifluoromethyl)sulfonyl]amide ([C_6_Meim][NTf_2_]) in a poly(vinyl chloride) matrix. Chronopotentiometry is utilized to determine the concentration of charge carriers, ionic diffusion coefficients and apparent dissociation constant of [C_6_Meim][NTf_2_] in PVC membranes plasticized with a mixture of [C_6_Meim][NTf_2_] and bis(2-ethylhexyl) sebacate (DOS) over a wide range of IL concentrations. The diffusion properties of [C_6_Meim][NTf_2_] are confirmed by NMR-diffusometry. The non-monotonic electrolytic behavior of the IL in PVC-DOS matrix is described for the first time. A maximum ionization degree and diffusion coefficient is observed at 30 wt.% of IL in the plasticizing mixture. Thus, it is shown that by varying the flexible parameter of the IL to plasticizer ratio in the polymeric phase one can tune the electrolytic and transport properties of sensing PVC membranes.

## 1. Introduction

Ionic liquids (ILs) are increasingly used in various fields of science. Good solvation properties, high ionic conductivity, non-volatility, relatively low toxicity, a large electrochemical window and good electrochemical stability make ILs promising organic solvents for studying electrochemical processes and for use in a number of analytical methods (electrochemical [1], chromatographic [2,3], etc.). Novel ion-selective, gas and biosensors with IL additives in the composition of sensing layers are being developed [4,5,6].

The use of ILs in the polymeric membranes of both potentiometric [7,8] and optical [9,10,11] sensors has been the focus of the chemical sensor community over the past decade, mainly due to their multifunctional performance. ILs can serve as active sensor components (ionophores and/or ionic additives) [8,12,13], at the same time plasticizing the polymeric sensing phase [7,14,15]. High stability and reproducibility of electrochemical sensor characteristics was achieved in [15] by using asymmetric imidazolium-based ILs in a double-functional mode: as a plasticizer and as an active component simultaneously. In [16], a PVC membrane doped with an imidazolium-based IL demonstrated a selective near-Nernstian response to sulfate ions over a broad concentration range, in the absence of other active components in the sensing layer. Moreover, stable and close to theoretical potentiometric responses can be obtained, even for membranes doped with ILs containing both cations and anions of relatively high lipophilicity [16], such as 1-butyl-2,3-dimethylimidazolium bis(trifluoromethylsulfonyl)amide ([BDMeim][NTf_2_]) and dodecylethyldiphenylphosphonium bis(trifluoromethylsulfonyl) amide ([DEDPP][NTf_2_]). The use of ILs as sensing components of electrochemical sensors is reviewed in [3,15]. Application of the ILs in the membranes of the related family of sensors, ion-selective polymeric optodes, is reviewed in [17].

An important area of IL application is the development of reference electrodes (REs) without a liquid junction [12,14,18] and of non-aqueous electrolyte bridges [19]. It is currently widely accepted that the distribution of an IL with close lipophilicities of the ions between aqueous and polymeric phases leads to stabilization of the Galvani potential difference at the membrane/solution interface regardless of the composition of the contacting solution. Disposable reference electrodes based on polymeric membranes doped with an IL were developed in [18] and showed stable electrode potential in real saliva samples for 24 h. In [12], solid state REs were created using ionic liquid and macroporous carbon as a transducer layer. The principles of developing IL-based liquid-junction free reference electrodes were formulated in one of the pioneering works [14]. The theoretical background to the stabilization of the interfacial potential when a lipophilic electrolyte is introduced into the sensing phase is given in [7,20]. Critical revision on the use of ILs for developing liquid-junction free REs and non-aqueous electrolyte bridges is comprehensively performed in [21].

One can notice that the available information on the behavior of polymeric membranes doped with ionic liquids is somewhat contradictory. On the one hand, the addition of ILs to the membranes of reference electrodes results in stabilization of their potential over a broad range of solutions, which is unequivocal evidence of ion exchange suppression at the membrane/solution interface. On the other hand, similar ILs are capable of improving the sensor characteristics of analogous membranes even when the lipophilicity of the cation and anion of the IL are close to each other. The authors of the respective reports use different concentrations of ILs depending on the purpose of the study. As a result, the behavior of the obtained membranes differs sharply, which may indicate abrupt and most likely non-monotonic dependence of the membrane properties on the IL content in the sensing layer. However, a systematic investigation of such a dependence over a broad range of IL concentrations in the membrane has not been reported thus far.

The ionic liquids being used as plasticizers change the viscosity and polarity of the polymeric phase as compared to the traditional plasticizers. Therefore, the ILs can heavily affect the ionic distribution coefficients, the stability of ion–ionophore complexes and the association/dissociation of ionic pairs in the sensing phase [22]. One can anticipate an increase in the polarity of the IL-containing media with the increase of the IL ionization degree; however, only indirect data is available in this regard. It was observed in [23] that the conductivity of some ionic liquids is significantly lower than the electrical conductivity of concentrated aqueous solutions due to a decrease in the number of available charge carriers caused by the formation of ion pairs. Meanwhile, in [24], it was shown that ionization of the IL leads to the decrease of viscosity of the studied systems. The dissociation constant of IL [C_6_Meim]Cl (0.385 mol/L), as well as the diffusion coefficient of [C_6_Meim]^+^ cation (7.65 × 10^−6^ cm^2^/s), were estimated in [25] for infinitely diluted aqueous solutions. A nearly 10-fold lower diffusion coefficient was measured in [26] for pure IL [C_2_Meim][NTf_2_] with NMR spectroscopy: 3 × 10^−7^ cm^2^/s to 9 × 10^−7^ cm^2^/s, depending on the temperature and applied electric field. Even lower diffusion coefficients, a relatively low dissociation constant (1.5 × 10^−3^ mol/L) and ionization degree of 0.59 were found in [27] for pure IL [C_6_Meim][NTf_2_]. Molecular dynamic simulations carried out for a binary mixture of [C_4_Meim][BF_4_] and series of solvents of various polarity showed that even in diluted solutions, IL predominately exists as cation/anion pairs provided that the solvent is low dielectric [28].

There is no available data on the electrolytic behavior of IL in polymers; however, the low polarity of PVC provides prerequisites for a significant association. Therefore, direct data on the ionization (or dissociation) degree of IL, especially on its dependence on the IL content in the polymeric matrix, is of particular interest.

In this work, we report a systematic electrochemical study of the electrolytic properties of IL in a poly(vinyl chloride) matrix. One of the most commonly used ionic liquid 1 hexyl-3-methyl-1H-imidazol-3-ium bis[(trifluoromethyl)sulfonyl]amide ([C6Meim][NTf2]) was studied as a plasticizing component in binary bis(2-ethylhexyl)sebacate ester-IL mixtures to plasticize PVC membranes. The resistivity of the resulting polymeric membranes, ionic diffusion coefficients, IL ionization degree and its apparent dissociation constant were studied in situ by means of chronopotentiometry and pulsed field gradient NMR diffusiometry (PFG-NMR) over a wide range of compositions of the plasticizing mixture.

## 2. Materials and Methods

High molecular weight poly(vinyl chloride) (PVC), bis(2-ethylhexyl) sebacate (DOS), volatile solvents: tetrahydrofuran (THF) and cyclohexanone (CH) were Selectophore grade reagents from Fluka (Buchs, Switzerland). 1-hexyl-3-methyl-1H-imidazol-3-ium bis[(trifluoromethyl)sulfonyl]amide ([C_6_Meim][NTf_2_]) was an HPLC (≥99.0%) reagent from Sigma Aldrich (St. Louis, MO, USA). KCl was an analytical grade reagent from Reakhim (Donetsk, Ukraine).

All aqueous solutions were prepared with deionized (DI) water with a resistivity of 18.2 MOhm × cm (Milli-Q Reference, Merck KGaA, Darmstadt, Germany).

Liquid membrane compositions were prepared by dissolving a weighed portion of PVC and an aliquot of a plasticizing mixture (IL and DOS) in CH or THF. The mass ratio of PVC to plasticizing mixture was kept constant at 1:3, which is a typical ratio for electrochemical and optical sensor membranes (1:3 to 1:2, [29]).

To obtain the membranes, the liquid compositions were stirred with a roller mixer Movil-Rod (J.P. Selecta, Barcelona, Spain) for 10 min and then cast on a Petri dish with diameter of 32 mm and closed with filter paper. After complete evaporation of the solvent (2 days in the case of THF and 7 days for CH), master membranes with a thickness of 0.2 mm were obtained (estimated with optical microscopy). After that, both series of membranes were cleaned from the accumulated exudate and weighed. The target compositions of the obtained membranes are listed in Table S1.

From the obtained master membranes, individual electrode membranes with a diameter of 8 mm were cut out. The cut membrane disks were glued to PVC bodies with an outer diameter of 8 mm and an inner diameter of 4 mm. A solution of PVC in CH was used as the glue (13 wt.%). Electrodes with 11 types of membranes were prepared, with three replicate electrodes of each membrane type. Before measurements, the electrodes were filled with 10^−3^ M KCl and conditioned in the same solution for 2 h (as in [7,14]). The internal reference electrode was electrochemically chlorinated silver wire.

Chronopotentiometric curves were recorded with Potentiostat-Galvanostat Autolab 302 N (Metrohm AG, Herisau, Switzerland). The measurements were performed in a conventional symmetric 3-electrode cell, filled with 10^−3^ M KCl, where the membrane electrode was the working electrode, Ag/AgCl electrode and glassy carbon rod served as reference and counter electrodes, respectively. In order to avoid convection effects and to provide diffusion control of the response, no stirring was applied. Electrochemical measurements were carried out in a Faraday cage to minimize background noise.

The open circuit potentials (OCPs) were recorded for the first 300 s, and then the electrode membranes were polarized for 60 s with a current pulse of 10^−8^ A and the potential was registered. After that, the current was turned off, and the potential was registered for another 60 s. The time resolution in chronopotentiometry measurements was 0.2 s. Appropriate corrections for solution resistance and for polarization of the Ag/AgCl electrode were performed by subtracting the chronopotentiometric curves obtained in the absence of the polymeric membrane in the otherwise same cell from the raw experimental data.

All electrochemical measurements were carried out in a plastic beaker with a volume of 15 mL, at room temperature: 24 ± 1 °C.

All electrochemical measurements were performed with 3 identical electrodes, and the obtained values were then averaged and statistically processed. To determine the resistivity and current density during chronopotentiometric measurements, the mean membrane surface area $\bar{S}$ was calculated through the harmonic average of the areas of the inner (*in*) and outer (*out*) surfaces in contact with the solution:

(1) $$\bar{S}=2/\left(1/S_{in}+1/S_{out}\right)$$

^1^H and ^19^F NMR measurements with pure [C_6_Meim][NTf_2_] and PVC-based membranes were carried out at 25 °C using a Bruker 400 WB NMR spectrometer (Bruker Corporation, Billerica, MA, USA) equipped with diffusion probe DIFF/50 with ^1^H and ^19^F coils and gradient controller GREAT MASTER UNIT E (Bruker Corporation, Billerica, MA, USA) with gradient amplifiers. The diffusion coefficients were measured by means of pulsed field gradient-stimulated echo with a bipolar gradient (PFG STEb technique) where the echo signal *I* is given by the well-known Stejscal-Tanner equation:

(2) $$I\left(G\right)=I_{0}\exp\left(-\gamma^{2}G^{2}\delta^{2}D\left(\Delta -\frac{\delta }{3}\right)\right),$$

with D: the diffusion coefficient, $I_{0}$: the integral intensity, γ: the gyromagnetic ratio, G: the gradient amplitude, δ: the equivalent duration of the gradient pulses, and Δ: the time interval between the gradient pulses. The following parameters were used: Δ = 20 ms, δ = 1.25 ms, the number of gradient steps was 64, and the number of scans was 16.

OriginPro 2015 (OriginLab Corporation, Northampton, MA, USA), MestReNova 14.2.1 (Mestrelab Research S.L., Santiago de Compostela, Spain), and MagicPlot PRO 2.9.3. (Magicplot Systems, LLC, St. Petersburg, Russia) were used for data processing.

## 3. Results and Discussion

### 3.1. NMR Studies of Pure IL [C_6_Meim][NTf_2_]

[C_6_Meim][NTf_2_] is one of the most commonly used ILs for developing ISEs [14] and optodes [17]. This IL is the most lipophilic among the commercially available ones; meanwhile, its ions are partially soluble in water [30,31]. Therefore, this IL is capable of producing a stable membrane boundary potential, which makes it a good candidate as a membrane electrolyte for developing liquid-junction free reference electrodes [14].

The electrolytic properties of pure IL were studied by NMR spectroscopy. The ^1^H NMR spectrum of pure [C_6_Meim][NTf_2_] is shown in Figure S1. The nature of imidazolium cation [C_6_Meim]^+^ and proton-free fluorine-containing anion [NTf_2_]^−^ allows separate determination of their diffusion coefficients by means of ^1^H and ^19^F NMR-diffusometry [26,27,32]. Equation (2) can be modified to obtain an expression that delivers the values of the diffusion coefficient directly from the experimental dependence of the integral intensity *I* on the square of gradient amplitude:

(3) $$I\left(x\right)=I_{0}\exp\left(-Dx\right);\;\mathrm{where}\;x=\gamma^{2}G^{2}\delta^{2}\left(\Delta -\frac{\delta }{3}\right)$$

Experimental dependencies $I\left(G\right)$ with ^1^H for the cation and with ^19^F for the anion were obtained. Each dependence was successfully fitted with a single value of the diffusion coefficient, which suggests complete ionization (dissociation) of pure IL. The resulting values (2.055 ± 0.006) × 10^−7^ cm^2^/s and (2.002 ± 0.013) × 10^−7^ cm^2^/s for the cation and anion, respectively, are consistent with the available literature data (2.2 × 10^−7^ cm^2^/s and 1.9 × 10^−7^ cm^2^/s for the cation and anion, respectively [27]).

### 3.2. Mechanical Properties of the IL-Doped PVC Membranes

**Figure 1:**
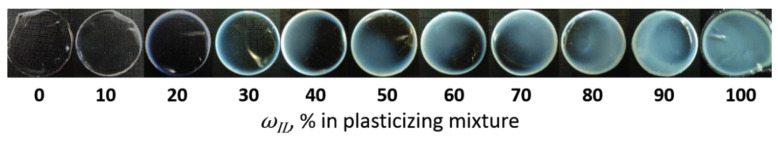
PVC membranes plasticized with a mixture of [C_6_Meim][NTf_2_] and DOS in various proportions.

According to [16], the use of an imidazolium-based IL as the only plasticizer for a PVC membrane does not provide an obvious advantage over traditional plasticizers, since it can negatively affect the mechanical properties of the membrane. However, the use of mixtures of traditional plasticizers with IL can potentially combine the advantages of both. Images of the obtained membranes containing a fixed amount of PVC and DOS-[C_6_Meim][NTf_2_] plasticizing mixtures varied over a broad range of IL fractions, are shown in Figure 1. The prepared samples included a control membrane plasticized with pure DOS with no added IL (0%) and a DOS-free membrane with 100 wt.% of IL in plasticizing mixture.

The elasticity of the obtained membranes decreased with the increase of the IL fraction in the plasticizing mixture. This observation corresponds to the data described in [16]. At the same time, starting from 20 wt.% of the IL in the plasticizing mixture, the dry membranes demonstrated slight opalescence, which increased with the growth of IL content.

Meanwhile, viscous transparent exudate was observed after removing the ready membranes from the Petri dish used for casting. In the case of the membranes cast from the CH solution, the amount of exudate was significantly higher. To identify the exudate, the dishes were rinsed with CCl_4_, and the obtained solution was analyzed using IR absorption spectroscopy. The exudate absorption bands (Figure S2) coincide with the spectral bands of pure [C_6_Meim][NTf_2_], except for the peak of relatively low intensity at the wave number of 1727 cm^−1^ typical for stretching of the C=O group in both esters and ketones [33]. Based on the additional spectra obtained in model mixtures containing both IL and a substance with a C=O bond (either DOS or CH), the peak at 1727 cm^−1^ was attributed to traces of the DOS plasticizer. It was shown earlier [34] by means of atomic force microscopy and time-of-flight static secondary ion mass spectroscopy that liquid-like films with a very low PVC/DOS ratio can form on the surface of plasticized PVC. It was shown that the surface of the relatively thick membranes, such as the studied ones, is more enriched with the plasticizer.

**Figure 2:**
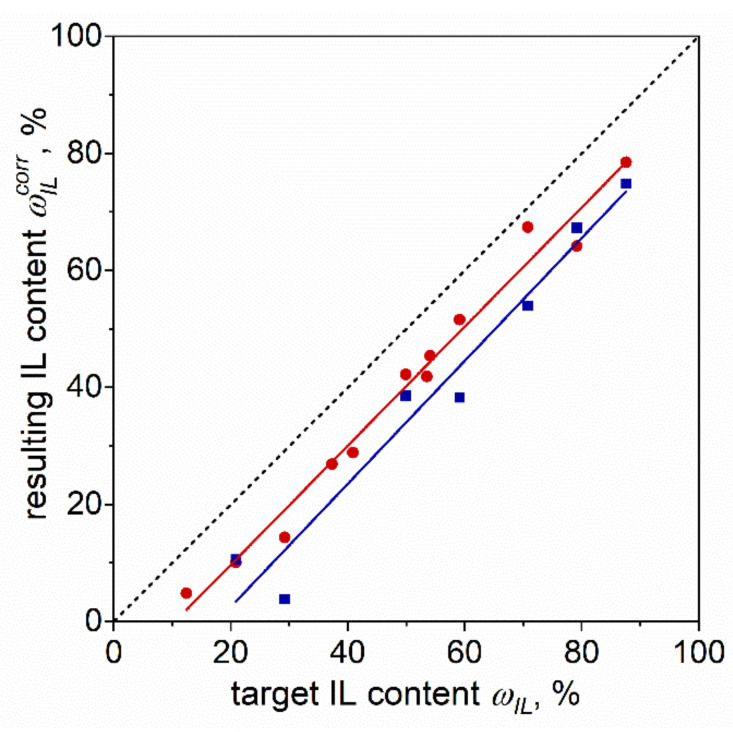
Correlation between the target ($\omega_{IL}$) and found ($\omega_{IL}^{corr}$) IL fraction in the plasticizing mixture. Squares—experimental data for the membranes cast from THF solution; circles—for the membranes cast from CH solution. Lines: linear fit; for THF: slope 1.02 ± 0.05, intercept −10.8 ± 2.4, r^2^ = 0.979; for CH: slope 1.05 ± 0.10, intercept −18.5 ± 6.1, r^2^ = 0.949.

The membranes were wiped from the exudate and weighed. The loss of the overall membrane mass was converted, according to the results of IR spectroscopy, into the loss in IL content; the results are shown in Figure 2 and in Table S2.

One can see from Figure 2 that the correlation between the found IL content and the target one is almost linear with a slope of 45°, and that exudation of the IL from the membrane is expressed in a parallel shift of the correlation line from the diagonal. This means that the average deviation of the resulting IL content, $\omega_{IL}^{corr}$, differs from the target one by a nearly constant value over the entire percentage range: ca. 10.8% and 18.5% in the case of THF and CH volatile solvents, respectively. The higher deviation in the case of CH as a solvent suggests that a longer evaporation time of a solvent and, consequently, a longer membrane drying period leads to a higher IL leaching rate. For this reason, further experiments were performed with the membranes cast from the THF-based liquid compositions.

### 3.3. Electrochemical and NMR Studies of IL-Doped Membranes

The specific electrical conductivity of an electrolyte is directly proportional to the concentration of charge carriers in it. Therefore, if the diffusion coefficients of ions of a given electrolyte are known, then the degree and constant of dissociation/association can be judged from the electrical conductivity. The diffusion coefficients of the ions can be measured independently with NMR-diffusometry [27,32]. Meanwhile, it was previously shown that it is possible to separately estimate the concentration and diffusion coefficient of charged species in a polymeric plasticized phase by means of chronopotentiometric measurements in a single-pulse galvanostatic mode [35,36].

**Figure 3:**
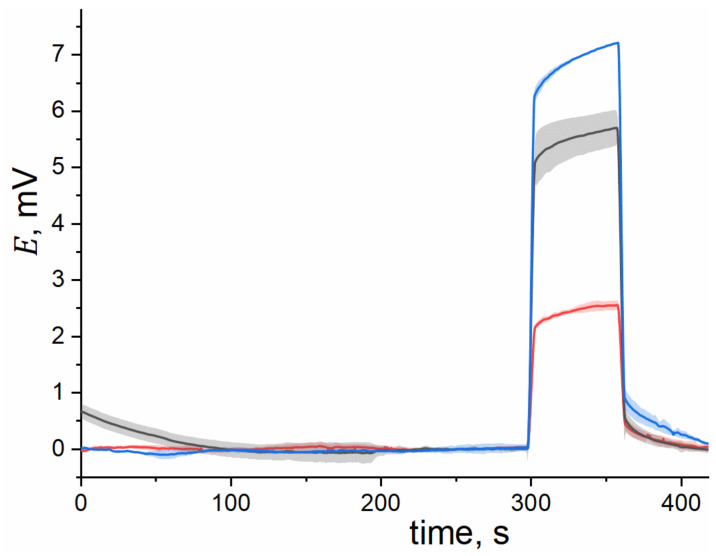
Chronopotentiometric curves obtained for electrode membranes containing 10 wt.% (black), 50 wt.% (red) and 80 wt.% (blue) of [C_6_Meim][NTf_2_] in the plasticizing mixture. Lines are averaged experimental data for 3 electrode membranes of identical composition. Due to the large number of data points, hereinafter the errors are represented as shaded area error bars for better clarity (OriginPro 2018 default function).

Electrodes containing different amounts of IL in the membrane (see Table S1) were polarized by direct current in a three-electrode symmetric cell containing 10^−3^ M KCl solution. Examples of chronopotentiometric curves recorded for the electrodes with 10, 50 and 80 wt.% of the IL in the plasticizing mixture in the membrane are shown in Figure 3.

During the first 300 s of measurement, before the polarizing current was turned on, the recorded potentials were nearly stable and close to 0 mV, due to the symmetry of the cell. At 300 s, the current was switched on, which caused an abrupt Ohmic voltage drop in the positive direction. The latter was followed by polarization accumulating over time. The interruption of current flow at 360 s resulted in a negative Ohmic drop followed by zero-current relaxation. In the case of polymeric plasticized membranes, the major contribution to the Ohmic potential drops is from the bulk resistance of the polymeric phase.

**Figure 4:**
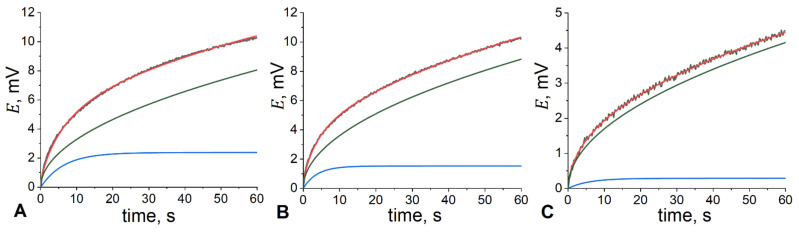
Polarization (**A**) and relaxation (**B**,**C**) domains of the chronopotentiometric curves obtained for electrode membranes containing 30 wt.% (**A**,**B**) and 70 wt.% (**C**) of [C_6_Meim][NTf_2_] in the plasticizing mixture. Both domains are plotted from (0;0) point, and the relaxation domain is inversed, for better clarity. Black (noisy) line: experimental data; red line: fitting with Equation (4); blue line: exponential contribution in Equation (4); green line: square root contribution in Equation (4). Fitting parameters: (**A**) A = 2.38 ± 0.04 mV, *τ* = 6.37 ± 0.13 s, B = 10.412 ± 0.011 mV·s^−1/2^; (**B**) A = 15.322 ± 0.010 mV, *τ* = 3.38 ± 0.08 s, B = 11.436 ± 0.012 mV·s^−1/2^; (**C**) A = 0.292 ± 0.021 mV, *τ* = 5.65 ± 0.64 s, B = 0.537 ± 0.004 mV·s^−1/2^.

The experimentally measured polarization curves, as well as zero-current relaxation curves, were fitted with the superposition of a decaying exponent and a square root function of time [35,36]:

(4) $$\eta =A\left(1-\exp\left(-t/\tau \right)\right)+B\sqrt{t}$$

where η stands for the polarization measured at time *t*, *τ* is the decay time of the exponent, and *A* and *B* are fitted parameters. An example of fitting of the experimental chronopotentiometric curve with Equation (4) and the respective contributions to the overall polarization/relaxation process are shown in Figure 4 for the electrode membrane with 30 and 70 wt.% of the IL in the plasticizing mixture.

Equation (4) corresponds to the total polarization process in the bulk of the membrane and at its boundaries with the solution. In the case of classical electrochemical systems, the first term $A\left(1-exp\left(-t/\tau \right)\right)$ corresponds to delayed charge transfer across the interface [35,36,37]:

(5) $$A\left(1-exp\left(-t/\tau \right)\right)=\eta_{\exp}=iR_{\mathrm{ct}}\left(1-exp\left(t/\tau \right)\right)$$

with *i*: the density of polarizing current, and $R_{\mathrm{ct}}$: the interfacial charge transfer resistance; however, transfer restrictions are usually not observed for polymeric membranes [29,35]. The polarizing current density is determined from the current pulse magnitude and the membrane area:

(6) $$i=I_{p}/\bar{S}$$

where $I_{p}$ is the polarizing current pulse, and $\bar{S}$ is the mean area of the membrane.

The second term $B\sqrt{t}$ is directly proportional to the root of time. According to the classical Delahay model [37], a linear dependence of the polarization over time at a constant current density indicates a transport-limited process. Therefore, the second term in Equation (4) can be attributed to the transport of charged species in the volume of the polymeric phase [35,36]:

(7) $$B\sqrt{t}=\eta_{\mathrm{diff}}=iR_{\mathrm{diff}}\sqrt{t}$$

where $R_{\mathrm{diff}}$ is the diffusional resistance which depends on *C_I_*: concentrations of the charged species and on *D_I_*: their mobilities in the polymeric phase according to the well-known equation [35,37]:

(8) $$R_{\mathrm{diff}}=\left(4RT/\sqrt{\pi }F^{2}\right)\left(\underset{I}{\sum }{\left(C_{I}\sqrt{D_{I}}\right)}^{-1}\right)$$

Thus, Equation (4) can be rewritten in the following shape:

(9) $$\eta =\eta_{\exp}+\eta_{\mathrm{diff}}=iR_{\mathrm{ct}}\left(1-exp\left(t/\tau \right)\right)+i\left(4RT/\sqrt{\pi }F^{2}\right)\left(\sum {\left(C_{I}\sqrt{D_{I}}\right)}^{-1}\right)\sqrt{t}$$

Generally, transport contribution to the overall measured polarization may involve both diffusion and possible migration of ions. However, in the case of relaxation curves registered after the current is already off, migration can be excluded from consideration. Meanwhile, the closeness of the fitted parameters obtained with Equation (4) from polarization and relaxation curves suggests a minor effect of migration in the transport contribution to the overall polarization.

Figure 4 demonstrates the adequacy of Equation (4) for describing the experimental data. The reduced χ^2^ values of fitting lay in a range of 10^−10^–10^−9^ (see Table S3 for specific numbers). The impact of the exponential process on the measured polarization was rather small; the decay times of the exponential contribution were in a range from 1 s to 7 s on average (see Table S3 for specific numbers), which suggests that the system remains mainly under diffusion control. The latter is in accordance with the generally accepted concept [29,35,36].

Using Equations (7) and (8), and the well-known relationship for resistivity [35,37], one can obtain the following equations for the case of the charge carriers *I* solely present in the membrane:

(10) $$\rho =RT/\left(2F^{2}C_{I}D_{I}\right)$$

(11) $$d\eta_{\mathrm{diff}}/d\sqrt{t}=8RTi/(F^{2}\sqrt{\pi }C_{I}\sqrt{D_{I}})$$

where *ρ* is the resistivity of the membrane, $\eta_{\mathrm{diff}}$ is the diffusion component of polarization (or relaxation), i is the density of the polarizing current, *C_I_* and *D_I_* are the concentration and mean diffusion coefficient of mobile charged species in the membrane. The value of resistivity can be calculated from the Ohmic drop in the bulk of the membrane, with a known geometry of the latter:

(12) $$\rho =R\left(\bar{S}/l\right)=(IR_{drop}/I)\left(\bar{S}/l\right)$$

where $IR_{drop}$ is the Ohmic drop, $\bar{S}$ is the mean area, and *l* is the thickness of the membrane.

To obtain both the volume concentration of charged species and their diffusion coefficients in the membrane, we shall assume that the ions of the IL are the only mobile charged species, and that their diffusion coefficients in the polymeric phase are close to the mean value:

(13) $$C_{{\left[C_{6}Meim\right]}^{+}}=C_{{\left[Ntf_{2}\right]}^{-}}=C_{I}/2$$

(14) $$D_{{\left[C_{6}Meim\right]}^{+}}≅D_{{\left[Ntf_{2}\right]}^{-}}≅\frac{D_{{\left[C_{6}Meim\right]}^{+}}+D_{{\left[Ntf_{2}\right]}^{-}}}{2}=D_{I}$$

The assumption expressed with Equation (14) is widely used for describing transport phenomena in the membrane phase [29,35,36]. In the case of [C_6_Meim][NTf_2_], this assumption is additionally justified by the closeness of the diffusion coefficients of cations and anions measured with NMR for pure ionic liquid: 2.055 × 10^−7^ cm^2^/s and 2.002 × 10^−7^ cm^2^/s, respectively (see Section 3.1). The simultaneous solution of Equations (10) and (11) leads to the expressions for the concentration of charged species and their mean diffusion coefficient in the membrane, respectively:

(15) $$C_{I}=i^{2}\rho \left(32RT/F^{2}\pi \right){\left(d\eta_{\mathrm{diff}}/d\sqrt{t}\right)}^{-2}$$

(16) $$D_{I}=i^{-2}\rho^{-2}\left(\pi /64\right){\left(d\eta_{\mathrm{diff}}/d\sqrt{t}\right)}^{2}$$

**Figure 5:**
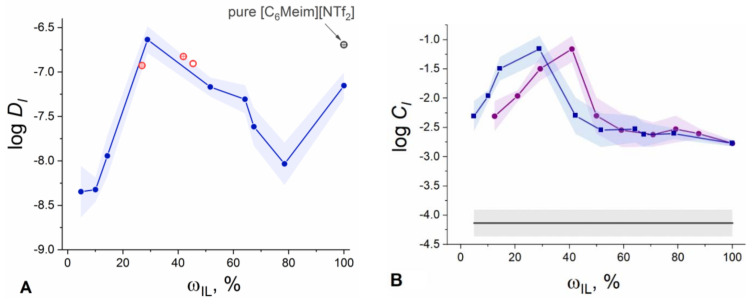
Dependence of (**A**) the mean diffusion coefficient (log *D_I_*) of charge carriers in the membrane vs. found content of IL; (**B**) concentration of IL ions (log *C_I_*) on target (circles) and found (squares) IL content in the membrane. Open symbols in panel (**A**): mean *D_I_* values obtained by NMR-diffusometry for pure [C_6_Meim][NTf_2_] (grey) and for IL-doped membranes (red). Horizontal line in panel (**B**): estimated level of ionic impurities in IL-free PVC-DOS membrane.

The values of *C_I_* and *D_I_* were estimated with Equations (15) and (16) using the obtained resistivities and $B=d\eta_{\mathrm{diff}}/d\sqrt{t}$: the fitting parameter in Equation (4). The dependencies of the concentrations of ions and of their mean diffusion coefficient on both the introduced ($\omega_{IL}$) and found ($\omega_{IL}^{corr}$) IL content in the membrane are shown in Figure 5. Table S3 summarizes the calculated logarithmic values of *C_I_* and mean *D_I_*.

**Figure 6:**
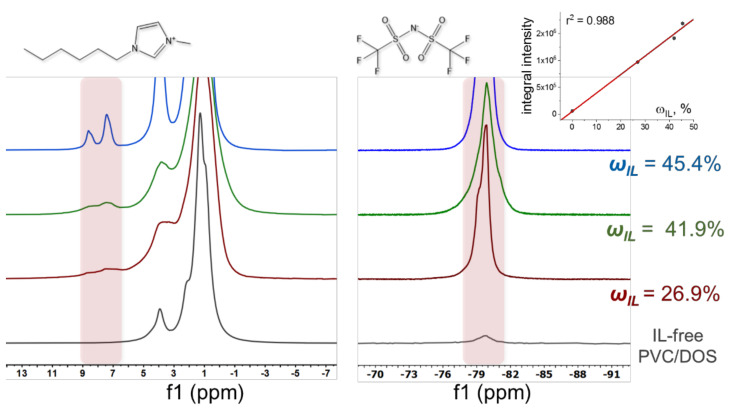
A stack of ^1^H (left panel) and ^19^F (right panel) spectra recorded for PVC-DOS membranes with different contents of [C_6_Meim][NTf_2_]: 0 wt.% (gray), 26.9 wt.% (red), 41.9 wt.% (green) and 45.4 wt.% (blue). Inset in the right panel: dependence of the integral intensity of the fluorine peak (−76 to −83 ppm) on the IL content (r^2^ = 0.988).

The values of diffusion coefficients were verified independently by NMR-diffusometry for the membranes containing 26.9, 41.9 and 45.4 wt.% of [C_6_Meim][NTf_2_] in plasticizing mixture after exudation of the IL excess. The results are summarized in Table S4, and the mean values of ionic diffusion coefficients are shown in Figure 5A with red open symbols. A stack of ^1^H and ^19^F spectra recorded for IL-containing PVC-DOS membranes, as well as for the control membrane with no [C_6_Meim][NTf_2_] added are shown in Figure 6. The spectral lines for all the samples are strongly broadened due to the presence of PVC-DOS matrix. In ^1^H spectra for the samples containing [C_6_Meim][NTf_2_], we observed a double peak between 6 and 9 ppm, which is characteristic of the protons of the imidazole ring of the IL cation. Moreover, the intensity of this peak grows with an increase of IL load in the membrane, while for the sample without IL it does not appear (gray line in Figure 6, left panel). The remaining peaks overlap with the peaks of the plasticizer protons (3–5.5 ppm and −2–3 ppm). A similar situation is observed in the ^19^F spectrum, which can be attributed to the fluorine-containing IL anion (Figure 6, right panel). In accordance with theoretical considerations, the integral intensity of the broadened peak of fluorine (−76 to −83 ppm) linearly increases with increasing IL content (inset in Figure 6, right panel).

^1^H and ^19^F spectra of the membrane with 45.4 wt.% of IL in the plasticizing mixture recorded at different values of the gradient amplitude are shown in Figure S3. Fitting the respective dependences *I*(*x*) with a single exponent (Equation (3)) gave unsatisfactory results; therefore, they were fitted with a superposition of the two decaying exponents [27,32]:

(17) $$I\left(x\right)=I_{1}\exp\left(-D_{1}x\right)+I_{2}\exp\left(-D_{2}x\right);\;\mathrm{with}\;x=\gamma^{2}G^{2}\delta^{2}\left(\Delta -\frac{\delta }{3}\right)$$

Each of the obtained exponents contained a “fast” component with a higher value of the fitting parameter *D*_1_, and a “slower” impact with a lower value of *D*_2_ (see Table S4 for specific numbers). The *D*_1_ values obtained from the ^1^H and ^19^F spectra differ from each other and can be attributed to the diffusion coefficients of the imidazolium cation and fluorine-containing anion, respectively. The “fast” component and the corresponding diffusion coefficient *D*_1_ values required for successful fitting of *I*(*x*) dependences for all three peaks in ^1^H spectra (6–9 ppm, 3–5.5 ppm and −2–3 ppm, Figure S3) coincided within the limits of error. Given that the peaks at 3–5.5 ppm and −2–3 ppm overlap with the DOS signal, this can serve as indirect evidence of the absence of the plasticizer’s impact on the estimated values of *D*_1_ diffusion coefficients.

Diffusion coefficients *D*_2_ for the second (“slow”) component of Equation (17) obtained for both cations and anions are equal within the margin of error (Table S4) and are almost an order of magnitude lower than the ionic diffusion coefficients: (5.33 ± 0.05) × 10^−8^ cm^2^/s. This result indicates that some amount of the IL diffuses in the form of an ion pair [32].

The mean values of ionic diffusion coefficients obtained by NMR (Figure 5A, red symbols) are in good agreement with the values obtained using chronopotentiometry. Both methods suggest the non-monotonic dependence of the diffusion coefficient versus IL content in the polymeric phase: the mean *D_I_* goes through a maximum between 20 and 40 wt.% of IL in the plasticizing mixture. The value of the mean diffusion coefficient, found for the membranes with ca. 30 wt.% of IL in the plasticizing mixture, reaches 2.3 × 10^−7^ cm^2^/s, which is slightly higher than our estimate for pure IL (ca. 2 × 10^−7^ cm^2^/s). Expectedly, in the membranes plasticized solely with the IL, the mean diffusion coefficient is significantly lower: 7.1 × 10^−8^ cm^2^/s.

The observed maximum of *D_I_* corresponds to the maximal values of ion concentration in the polymeric phase (Figure 5B). This behavior readily correlates with the typical shape of dependencies of the specific conductivity versus concentration for conventional aqueous electrolytes. For the concentration of charge carriers, a maximum is observed at 30 wt.% of IL in the plasticizing mixture, suggesting a maximal ionization degree in this concentration range (Figure 5B). The correlation between *D_I_* and *C_I_* in the polymeric membrane is consistent with the report on an increase in ionic mobilities due to a decrease in viscosity with growing ionization of the IL [24].

The estimated concentration of ions in the membranes without IL corresponds well with the literature data on the content of ionic impurities in PVC-DOS membranes; compare 79 μmol/L obtained in this study, 53 and 73 μmol/L obtained in [38] and 103 μmol/L obtained in [39].

The observed dependencies can be further developed into an explanation for the non-monotonic change in the plasticizing and sensing properties of ILs in the compositions of polymeric membranes described in the literature. Moreover, the obtained data allows for deliberately influencing the electrolytic and transport behavior of IL in the polymeric phase through a flexible parameter of IL to plasticizer ratio.

The IL ionization (dissociation) degree $\alpha_{\left[C_{6}Meim\right]\left[Ntf_{2}\right]}$ was calculated as follows:

(18) $$\alpha_{\left[C_{6}Meim\right]\left[Ntf_{2}\right]}=C_{I}/\left(2C_{IL}^{corr}\right)$$

where *C_I_* is the concentration of charged species calculated with Equation (15), $C_{IL}^{corr}$ is the total volume concentration of the ionic liquid in the membrane corrected to the IL loss during preparation. The data on the dissociation of the IL, the resulting IL volume concentrations and ionization degree values are collected in Table S5.

**Figure 7:**
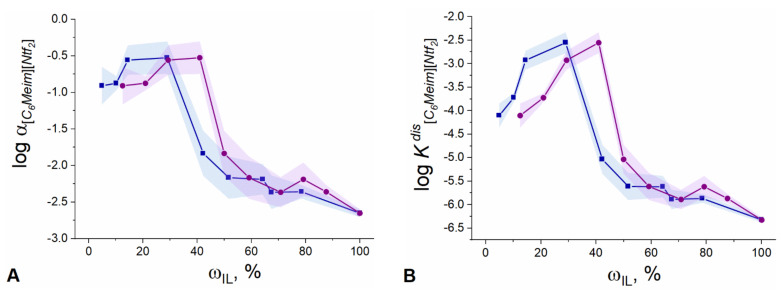
(**A**) Dependence of ionization degree of [C_6_Meim][NTf_2_] (in log units) on target (circles) and found (squares) IL content in the membrane. (**B**) Dependence of the dissociation constant of [C_6_Meim][NTf_2_] (in log units) in PVC membrane on target (circles) and found (squares) IL content in the membrane.

The dependence of [C_6_Meim][NTf_2_] ionization degree on the introduced and resulting IL content is shown in Figure 7A.

The dependences shown in Figure 7A follow the same non-monotonic trend as observed before (see Figure 5), with a maximum at 30 wt.% of IL in the plasticizing mixture, where the value of $\alpha_{\left[C_{6}Meim\right]\left[Ntf_{2}\right]}$ reaches nearly 0.32 (Table S5, Figure 7A). Apparently, around 20–30 wt.% of IL content in the plasticizing mixture, steric restrictions on ionization of IL are removed to the maximum extent due to the presence of a liquid plasticizer in a rigid PVC matrix. At the same time, the values of $\alpha_{\left[C_{6}Meim\right]\left[Ntf_{2}\right]}$ (Table S5) generally indicate a relatively weak dissociation which is consistent with the data reported in [28] on electrolytic behavior of similar ILs in the solvents of low polarity (dielectric constant for DOS is 3.9 [29]). A significant amount of [C_6_Meim]^+^[NTf_2_]^−^ ion pairs with the diffusion coefficient of (5.33 ± 0.05) × 10^−8^ cm^2^/s formed in the polymeric phase, is confirmed by NMR data (see above).

With an increase of the IL fraction above 30 wt.% relative to the plasticizer, a tremendous decrease in ionization degree is observed. The difference between maximal values of $\alpha_{\left[C_{6}Meim\right]\left[Ntf_{2}\right]}$ and the values obtained for the membranes plasticized with pure IL is more than two orders of magnitude (Figure 7A). This can be explained by accumulating deficiency of the liquid solvent: DOS, upon the increase of the relative IL content in the plasticizing mixture. As a result, the fraction of ion pairs in IL increases.

The apparent dissociation constant of [C_6_Meim][NTf_2_] in PVC membranes was calculated according to Equation (19):

(19) $$K_{\left[C_{6}Meim\right]\left[Ntf_{2}\right]}^{dis}={\left(C_{I}/2\right)}^{2}/\left(C_{IL}^{corr}-C_{I}/2\right)$$

where *C_I_* is the concentration of charged species calculated with Equation (15), $C_{IL}^{corr}$ is the total volume concentration of the ionic liquid in the membrane corrected to the IL loss during manufacture. The dependence of the mean logarithm of the $K_{\left[C_{6}Meim\right]\left[Ntf_{2}\right]}^{dis}$ on both introduced and found IL content in the membrane is shown in Figure 7B.

According to Figure 7B, the dependence of the dissociation constant of [C_6_Meim][NTf_2_] on the IL content in the membrane goes through a maximum, delivering a value of $K_{\left[C_{6}Meim\right]\left[Ntf_{2}\right]}^{dis}$ as high as 2.8 × 10^−3^ mol/L at 30 wt.% of the ionic liquid in the plasticizing mixture. At a higher IL content, there is a general trend toward a decrease of the IL apparent dissociation constant. The values of $K_{\left[C_{6}Meim\right]\left[Ntf_{2}\right]}^{dis}$ for the entire span of IL concentrations cover the range from −2.6 to −6.3 in log units.

The results shown in Figure 7B suggest that the electrolytic properties of the IL are characterized not only by a degree of ionization, but also by the value of the apparent dissociation constant. The strong dependence of the latter on the IL concentration in the polymeric phase may indicate changes in the overall solvation properties of the medium with varying IL content due to e.g., a change in polarity, and/or in the interplay between PVC and DOS in the respective binary subsystem [29].

## 4. Conclusions

In this work, PVC membranes doped with mixtures of bis(2-ethylhexyl) sebacate and IL 1-hexyl-3-methyl-1H-imidazol-3-ium bis[(trifluoromethyl)sulfonyl]amide were studied by means of chronopotentiometry and NMR-diffusometry. The dependences of the resistance of the fabricated membranes, ionic diffusion coefficients and concentration of charged species in the polymeric phase on IL fraction were obtained in a broad range of IL concentrations. The values of ionic diffusion coefficients were verified and the diffusion coefficient for the ion pair [C_6_Meim]^+^[NTf_2_]^−^ was evaluated with the PFG-NMR technique. Ionization degree and apparent dissociation constant of the studied IL were determined in situ in the polymeric phase for varied membrane compositions; the respective values were from 0.0012 to 0.316 and from 4.7 × 10^−7^ mol/L to 2.8 × 10^−3^ mol/L for the ionization degree and dissociation constant, respectively. The non-monotony of the electrolytic properties of the IL in the polymeric phase was demonstrated for the first time.

Based on the obtained results, we conclude that by varying the relative content of IL in the polymeric phase, it is possible to influence the properties of sensing PVC membranes, namely their conductivity, viscosity and polarity. The observed dependences pave the way for further explanation of the effect of ILs on the response of potentiometric and other polymeric sensors, in particular, non-monotonic changes in the plasticizing and sensing properties of ILs added to polymeric membranes. Importantly, the revealed regularities can be applied for tailoring the electrolytic and transport behavior of IL in the polymeric phase through a flexible parameter of the IL to plasticizer ratio in order to improve the required characteristics of a sensor.

## Data Availability

The data are available upon reasonable request from the corresponding author.

## Supplementary Materials

The following are available online at https://www.mdpi.com/article/10.3390/membranes12020130/s1, Table S1: Composition of the prepared PVC-[C_6_Meim][NTf_2_]-DOS membranes; Figure S1: ^1^H NMR spectrum of pure IL [C_6_Meim][NTf_2_]; Figure S2: IR absorption spectra of pure [C_6_Meim][NTf_2_], membrane exudate, and [C_6_Meim][NTf_2_]-DOS mixture (ω_DOS_ = 5%); Table S2: Recalculated IL content in the membranes cast from THF- and CH-based liquid compositions; Table S3: The data obtained from fitting of the chronopotentiometric curves registered with PVC membranes doped with various amount of [C_6_Meim][NTf_2_]; Table S4: Diffusion coefficients obtained from fitting integral intensities in ^1^H and ^19^F NMR spectra registered with PVC membranes doped with various amounts of [C_6_Meim][NTf_2_], versus square of the gradient amplitude (*x*); *D*_1_—“fast” component, *D*_2_—“slow” component in Equation (17); Figure S3: ^1^H and ^19^F spectra of the membrane with 45.4 wt.% of IL in the plasticizing mixture at different values of the gradient amplitude. Insets: the respective experimental and fitted dependences of the integral intensity on the square of gradient amplitude (*x*) for ^1^H and ^19^F spectra; Table S5: The target and found IL concentrations in the studied membranes and the respective values of [C_6_Meim][NTf_2_] ionization degree.
