# Supplementary material for: Chronopotentiometric Evaluation of Ionization Degree and Dissociation Constant of Imidazolium-Based Ionic Liquid [C6Meim][NTf2] in Polymeric Plasticized Membranes
Source: Membranes (Basel). 2022 Jan 21;12(2):130. doi: 10.3390/membranes12020130 (PMC8877390; doi:10.3390/membranes12020130)
Supplement: Supplementary file 1 [file membranes-12-00130-s001.zip › membranes-1558600-supplementary.pdf]

membranes

Supplementary Materials

Chronopotentiometric Evaluation of Ionization Degree and Dissociation Constant of Imidazolium-Based Ionic Liquid [C_6Meim][NTf_2] in Polymeric Plasticized Membranes

Nadezhda V. Pokhvishcheva [1], Elizaveta K. Gigiadze [1], Andrey V. Kalinichev [1], Alexandr V. Ievlev [2], Konstantin V. Tyutyukin [2] and Maria A. Peshkova [1,*]

[1] Institute of Chemistry of Saint Petersburg State University, 7/9 Universitetskaya nab, 199034 St. Petersburg, Russia; st040584@student.spbu.ru (N.V.P.); st055887@student.spbu.ru (E.K.G.); a.v.kalinichev@spbu.ru (A.V.K.)

[2] Faculty of Physics of Saint Petersburg State University, 7/9 Universitetskaya nab, 199034 St. Petersburg, Russia; a.ievlev@spbu.ru (A.V.I.); k.tyutyukin@spbu.ru (K.V.T.)

* Correspondence: m.peshkova@spbu.ru

Citation: Pokhvishcheva, N.V.; Gigiadze, E.K.; Kalinichev, A.V.; Ievlev, A.V.; Tyutyukin, K.V.; Peshkova, M.A. Chronopotentiometric Evaluation of Ionization Degree and Dissociation Constant of Imidazolium-Based Ionic Liquid [C_6Meim][NTf_2] in Polymeric Plasticized Membranes. *Membranes* **2022**, *12*, 130. https://doi.org/10.3390/membranes12020130

Academic Editors: Beata Paczosa-Bator and Amir Razmjou

Received: 31 December 2021
Accepted: 18 January 2022
Published: 21 January 2022

Publisher's Note: MDPI stays neutral with regard to jurisdictional claims in published maps and institutional affiliations.

Copyright: © 2022 by the authors. Licensee MDPI, Basel, Switzerland. This article is an open access article distributed under the terms and conditions of the Creative Commons Attribution (CC BY) license (https://creativecommons.org/licenses/by/4.0/).

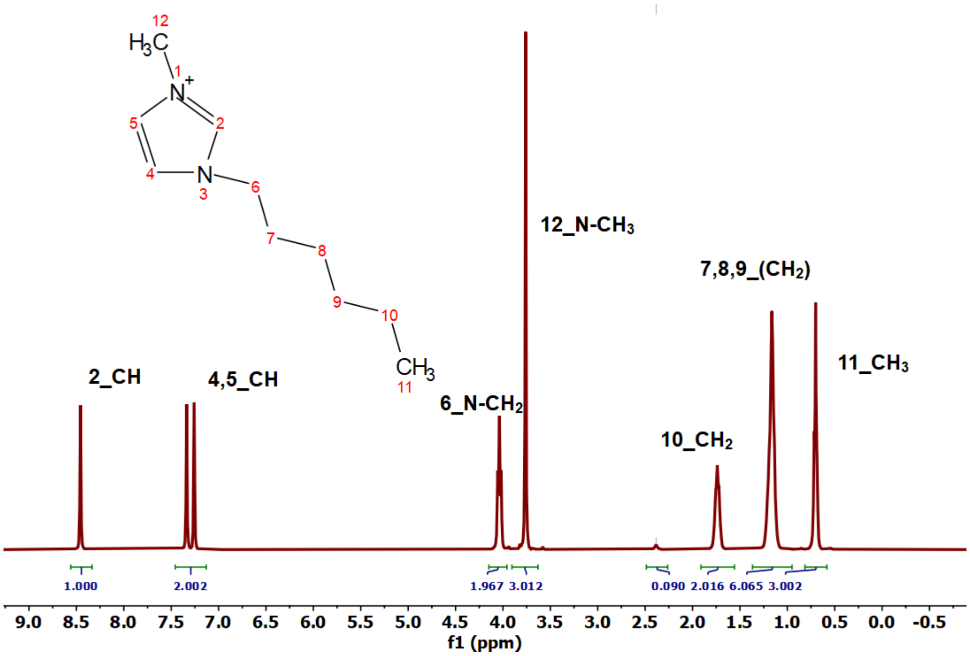

Figure S1. ^{1}H NMR spectrum of pure IL [C_6Meim][NTf_2].

Table S1. Composition of the prepared PVC−[C_6Meim][NTf_2]−DOS membranes.

<table>
<tr><td colspan="2">Target IL Content (ω_{IL}, % in plasticizing mixture)</td><td>0</td><td>12.4</td><td>20.8</td><td>29.2</td><td>37.3</td><td>40.9</td><td>50</td><td>53.5</td><td>54.1</td><td>59.1</td><td>70.8</td><td>79.2</td><td>87.6</td><td>100</td></tr>
<tr><td colspan="2">Target IL Content (C_{IL}, mol/L of the membrane volume)</td><td>0</td><td>0.211</td><td>0.351</td><td>0.494</td><td>0.634</td><td>0.691</td><td>0.840</td><td>0.907</td><td>0.916</td><td>0.999</td><td>1.195</td><td>1.332</td><td>1.487</td><td>1.687</td></tr>
<tr><td rowspan="3">Membrane Composition</td><td>IL, (mg)</td><td>0</td><td>23.5</td><td>38</td><td>54.1</td><td>71</td><td>76.1</td><td>91.1</td><td>100.4</td><td>101.4</td><td>110</td><td>131</td><td>144.3</td><td>165.8</td><td>184.4</td></tr>
<tr><td>DOS, (mg)</td><td>184.4</td><td>165.8</td><td>144.3</td><td>131</td><td>119.3</td><td>110</td><td>91.4</td><td>87.1</td><td>86</td><td>76.1</td><td>54.1</td><td>38</td><td>23.5</td><td>0</td></tr>
<tr><td>PVC, (mg)</td><td colspan="14">60</td></tr>
</table>

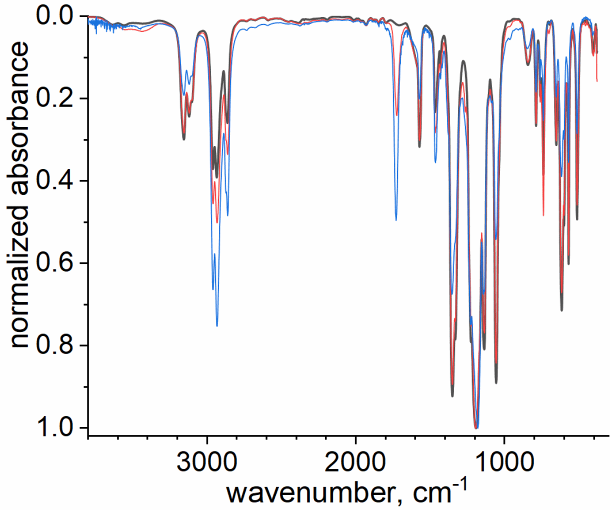

Figure S2. IR absorption spectra of pure [C_6Meim][NTf_2] (black), membrane exudate (red), and [C_6Meim][NTf_2]−DOS mixture (ω_{DOS} = 5%, blue).

Table S2. Recalculated IL content in the membranes cast from THF- and CH-based liquid compositions.

Target IL Content (ω_{IL}, % in plasticizing mixture)		0	12.4	20.8	29.2	37.3	40.9	49.9	53.5	54.1	59.1	70.8	79.2	87.6	100
**Found IL Content** (ω_{IL}, % in plasticizing mixture)	THF	0	4.8	10.1	14.4	26.9	28.9	42.2	41.9	45.4	51.6	67.4	64.2	78.5	-
	CH	0	outlier	10.6	3.8	-	outlier	38.6	-	-	38.3	54	67.3	74.8	-

Table S3. The data obtained from fitting chronopotentiometric curves registered with PVC membranes doped with various amount of $[C_6Meim][NTf_2]$, with Equation 4.

**Target IL Content** (ω_{IL}, % in plasticizing mixture)	**Found IL Content** (ω_{IL}, % in plasticizing mixture)	mean $\chi^2 \times 10^9$	mean τ, (s)	**log C_I**	**log D_I**
0	0	-	-	−4.1 ± 0.2	-
12.4	4.8	0.98	1.6	−2.3 ± 0.3	−8.35 ± 0.29
20.8	10.1	1.9	3.7	−2.0 ± 0.1	−8.32 ± 0.14
29.2	14.4	3.1	3.4	−1.5 ± 0.2	−7.94 ± 0.23
40.9	28.9	3.9	3.7	−1.2 ± 0.2	−6.63 ± 0.15
49.9	42.2	7.3	4.7	−2.3 ± 0.3	−6.92 ± 0.17
59.1	51.6	4.9	2.8	−2.5 ± 0.3	−7.17 ± 0.11
70.8	64.2	4.2	4.9	−2.6 ± 0.2	−7.31 ± 0.16
79.2	67.4	3.4	6.6	−2.5 ± 0.2	−7.62 ± 0.21
87.6	78.5	2.9	4.7	−2.6 ± 0.1	−8.03 ± 0.24
100	100	2.2	5.9	−2.8 ± 0.1	−7.15 ± 0.15

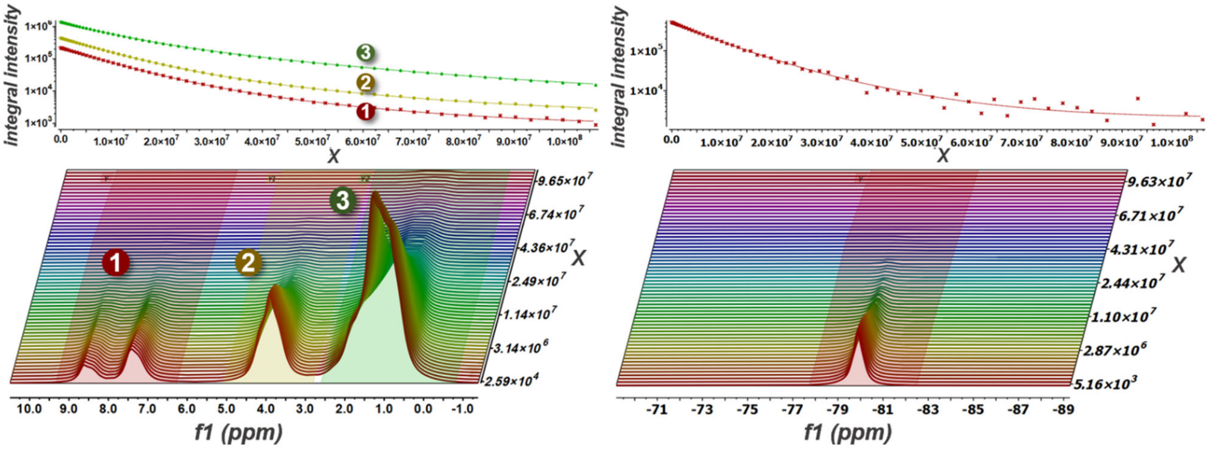

Figure S3. A stack of ^{1}H (left panel) and ^{19}F (right panel) spectra of the membrane with 45.4 wt.% of IL in the plasticizing mixture at different values of the gradient amplitude. Insets: the respective experimental (symbols) and fitted with Equation 117 (lines) dependences of the integral intensity on the square of gradient amplitude (x) for ^{1}H and ^{19}F spectra.

Table S4. Diffusion coefficients obtained from fitting integral intensities in ^1H and ^{19}F NMR spectra registered with PVC membranes doped with various amount of [C_6Meim][NTf_2], versus square of the gradient amplitude (x); D_1—"fast" component, D_2—"slow" component in Equation 17.

**Found IL Content** (ω_{IL}, % in plasticizing mixture)	obtained from cation peaks in ^1H spectra, × 10^7 cm^2/s		obtained from anion (fluorine) peaks in ^{19}F spectra, × 10^7 cm^2/s		**mean D_1**, log units	**mean D_2**, log units
	D_1	D_2	D_1	D_2		
26.9	1.108 ± 0.011	0.574 ± 0.009	1.285 ± 0.018	0.498 ± 0.081	−6.928 ± 0.006	−7.272 ± 0.044
41.9	1.731 ± 0.008	0.558 ± 0.009	1.295 ± 0.012	0.531 ± 0.024	−6.825 ± 0.004	−7.264 ± 0.016
45.4	1.192 ± 0.008	0.446 ± 0.021	1.303 ± 0.074	0.592 ± 0.142	−6.905 ± 0.025	−7.290 ± 0.087
pure [C_6Meim][NTf_2]	2.055 ± 0.006	-	2.002 ± 0.013	-	-	-

Table S5. The target and found IL concentrations in the studied membranes, and the respective values of [C_6Meim][NTf_2] ionization degree.

**Target IL Content**	**Found IL Content**		**ionization degree, $\alpha_{[C_6Meim][Ntf_2]}$**
**ω_{IL}, % in plasticizing mixture**	**ω_{IL}^{corr}, % in plasticizing mixture**	**C_{IL}, mol/l of the membrane volume**	
12.4	4.8	0.080	0.13 ± 0.09
20.8	10.1	0.164	0.13 ± 0.03
29.2	14.4	0.231	0.25 ± 0.12
40.9	28.9	0.466	0.32 ± 0.15
49.9	42.2	0.685	(16 ± 12) × 10^{-3}
59.1	51.6	0.836	(6 ± 5) × 10^{-3}
70.8	67.4	1.107	(4.0 ± 1.9) × 10^{-3}
79.2	64.2	0.917	(6 ± 3) × 10^{-3}
87.6	78.5	1.133	(4.0 ± 1.9) × 10^{-3}
100	100	1.518	(1.2 ± 0.5) × 10^{-3}
